# Imprecision and Preferences in Interpretation of Verbal Probabilities in Health: a Systematic Review

**DOI:** 10.1007/s11606-021-07050-7

**Published:** 2021-08-06

**Authors:** Katerina Andreadis, Ethan Chan, Minha Park, Natalie C Benda, Mohit M Sharma, Michelle Demetres, Diana Delgado, Elizabeth Sigworth, Qingxia Chen, Andrew Liu, Lisa Grossman Liu, Marianne Sharko, Brian J Zikmund-Fisher, Jessica S Ancker

**Affiliations:** 1 Department of Population Health Sciences, Weill Cornell Medicine, New York, NY USA; 2 Memorial Sloan Kettering Cancer Center, New York, NY USA; 3 Samuel J Wood Library, Weill Cornell Medicine, New York, NY USA; 4 Department of Biostatistics, Vanderbilt University Medical Center, Nashville, TN USA; 5 Department of Health Behavior and Health Education, University of Michigan School of Public Health, Ann Arbor, MI USA; 6 Department of Biomedical Informatics, Columbia University Vagelos School of Medicine, New York, NY USA; 7 Department of Biomedical Informatics, Vanderbilt University Medical Center, Nashville, TN USA

**Keywords:** risk communication, patient-provider communication, health literacy, health numeracy

## Abstract

**Introduction:**

Many health providers and communicators who are concerned that patients will not understand numbers instead use verbal probabilities (e.g., terms such as “rare” or “common”) to convey the gist of a health message.

**Objective:**

To assess patient interpretation of and preferences for verbal probability information in health contexts.

**Methods:**

We conducted a systematic review of literature published through September 2020. Original studies conducted in English with samples representative of lay populations were included if they assessed health-related information and elicited either (a) numerical estimates of verbal probability terms or (b) preferences for verbal vs. quantitative risk information.

**Results:**

We identified 33 original studies that referenced 145 verbal probability terms, 45 of which were included in at least two studies and 19 in three or more. Numerical interpretations of each verbal term were extremely variable. For example, average interpretations of the term “rare” ranged from 7 to 21%, and for “common,” the range was 34 to 71%. In a subset of 9 studies, lay estimates of verbal probability terms were far higher than the standard interpretations established by the European Commission for drug labels. In 10 of 12 samples where preferences were elicited, most participants preferred numerical information, alone or in combination with verbal labels.

**Conclusion:**

Numerical interpretation of verbal probabilities is extremely variable and does not correspond well to the numerical probabilities established by expert panels. Most patients appear to prefer quantitative risk information, alone or in combination with verbal labels. Health professionals should be aware that avoiding numeric information to describe risks may not match patient preferences, and that patients interpret verbal risk terms in a highly variable way.

**Supplementary Information:**

The online version contains supplementary material available at 10.1007/s11606-021-07050-7.

## INTRODUCTION

Although probabilities of risk and benefit are often important components of medical information, it is well established that many patients have low numeracy, which may impair their ability to understand or make decisions on the basis of numerical information.^1–5^ As a result, healthcare providers may believe that patients will not be able to use numerical information or that they prefer words to numbers.^6^ In one survey, only about 35% of ob-gyns reported routinely using numbers when talking with patients about screening tests, with the remainder preferring verbal terms such as “low risk” or labels such as “normal/abnormal.”^7^ A different survey found that family physicians used numbers or quantitative graphics to describe cardiovascular risk in only 27% of patient visits; in the remainder of the visits, the physician used verbal risk terms only.^8^ Healthcare providers’ use of quantitative risk information (in the form of numbers or graphics) appears to be related to factors including their own numeracy, their perception of the patients’ numeracy, and the gender of both the provider and the patient.^8,9^

In non-medical domains, risk communication research has demonstrated that a major limitation of relying on verbal probability terms is that they are interpreted in highly variable ways by the recipients of the information.^10–13^ An additional source of uncertainty in verbal risk communication is that the speaker may choose different verbal probability terms according to their opinion and previous experiences, and this choice is likely to in turn influence the recipient’s judgment.^14^ One study found that choice of verbal terms is even influenced by politeness, so that polite speakers generally communicated lower risk magnitudes than less polite ones.^15^

However, extrapolating findings from non-medical contexts to medical ones may be problematic, given the domain-specific nature of risk perceptions and behaviors.^10,16^ We therefore consider it important to assess the impact of verbal probability expressions in medical and health contexts only. Also, in light of healthcare professionals’ persistent use of verbal-only risk communications, we believe it is important to clarify whether patients in fact prefer verbal descriptions of risk to numerical ones.

Therefore, the objective of this study was to review the existing literature to synthesize evidence on patient interpretation of and preference for verbal probabilities in health and medical communication.

## METHODS

This study analyzed a subset of articles from a large systematic review of experimental and quasi-experimental research contrasting different formats (numerical, graphical, and verbal) for presenting health-related quantitative information to the lay public. The review included both probabilities (such as health risks) and quantities (such as laboratory values and environmental data) and was limited to studies measuring quantitative outcomes including preference, comprehension, and decisions.

**Figure 1:**
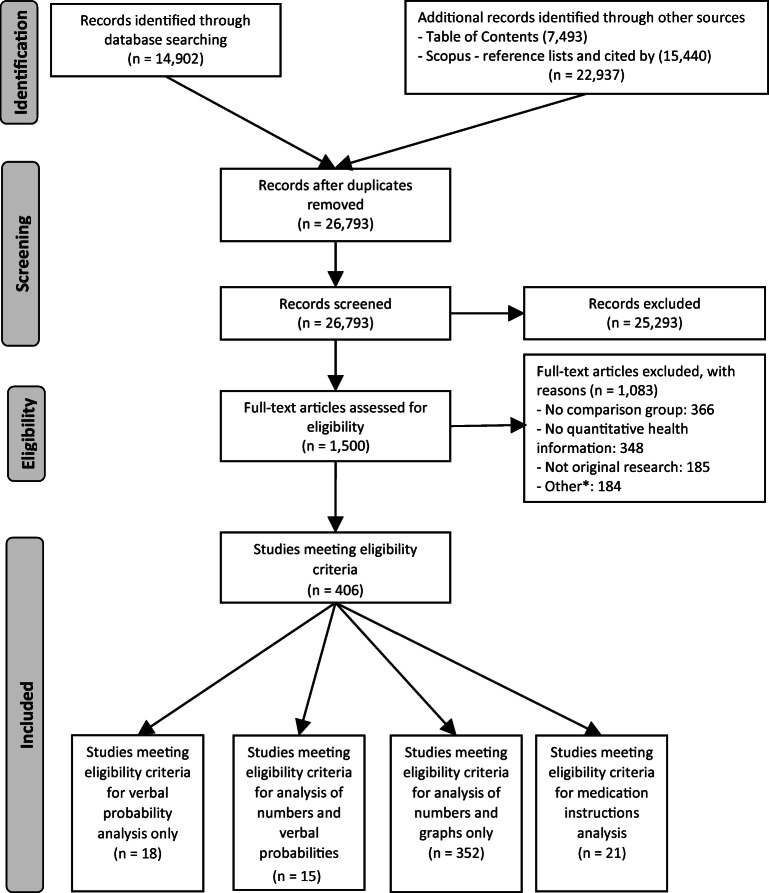
PRISMA flow diagram. *Other: duplicate dataset, no quantitative evaluation metric, insufficient detail to extract, not adults, experiments designed to understand beliefs not response to information, no full text available, test of education method, scale development/calibration, decision was not a personal health/medical decision, non-patient (health professional), comparator was different terms for cancer not different formats, verbal probabilities not in English.

We performed the systematic review following the Preferred Reporting Items for Systematic Reviews and Meta-Analyses (PRISMA) statement (Fig. 1).^17^ In adherence to these guidelines, we registered a protocol in PROSPERO (registration #CRD42018086270). Two experienced librarians constructed a systematic approach to search Ovid MEDLINE, Ovid Embase, the Cochrane Library (Wiley), CINAHL (EBSCO), ERIC (ProQuest), PsycINFO (EBSCO), and the ACM Digital Library, from inception to January 2019, with an update on September 10, 2020. See Appendix 1 for the search strategy for Ovid MEDLINE. To supplement these results, we identified the top 4 most common journals from database searches (*Medical Decision Making*, *Patient Education and Counseling*, *Risk Analysis*, and *Journal of Health Communication*) and hand-searched their tables of contents in their entirety from 2008 up to 2019. For articles selected for inclusion in this study, we pulled and screened reference lists and citing articles from Scopus (Elsevier). Searches produced a total of 37,839 articles. After de-duplication, two independent reviewers screened 26,793 titles and abstracts using Covidence systematic review web software (Covidence.org, Melbourne, Australia). We then assessed 1500 articles for full-text review, with discrepancies resolved by consensus or third reviewer.

Pairs of reviewers performed full-text review. A three-member verbal probabilities team (KA, EC, MP) reviewed all articles studying verbal probability terms such as “rare,” “common,” or “likely” (76 articles) and included publications meeting the following criteria:

1. Original studies presenting participants with health-related information.
2. Sample was adult laypeople without expertise in a health profession (the study was included if at least one subsample met these inclusion criteria and if the results for the subsample were reported separately).
3. Quantitative assessments of outcomes included:
  - Numerical estimates of the meaning of the verbal term, and/or
  - Preferences for verbal vs numeric terms.
4. Verbal probabilities expressed in English. (The larger review did not include a language restriction. However, for this analysis of verbal probabilities, we limited research to studies conducted in English to ensure that interpretations of the verbal terms would not be confounded by potential differences in translation.)

Three reviewers extracted data from the included articles using a custom-developed Qualtrics instrument. Data extracted included the main question or comparison, the outcomes measured (either numerical interpretation of the probability terms, preferences, or both), the sample size, and the population recruited. We captured details about the stimuli including the specific verbal terms studied; whether the probability was chance of disease, medication side effects, or adverse effects of a procedure; the general health condition or domain if specified; and the severity of the health event if specified, e.g., mild or severe.

In recording the outcome of numerical estimates of a verbal probability, we recorded sample sizes, mean estimates, ranges, and (where provided) either standard deviations or 95% confidence intervals. In the list of verbal terms, we did not distinguish between adjectival and adverbial forms (e.g., *rare* and *rarely*). A subset of studies examined the terms listed in the European Commission (EC) guidelines, which standardize the use of verbal probability terms to be used for medication side effects.^18^ (For example, these guidelines specify that the term “rare” is to be used for events with probabilities between 0.01 and 0.1%, and the term “very rare” for probabilities lower than < 0.01%.^18^) For these studies, we also recorded a “correct/incorrect” flag by whether respondents provided a numeric probability within the range specified by the EC for that term. In recording the outcome of preference, we recorded the proportions who reported preferring words, numbers, both, other, or no preference.

To pool the estimated probabilities from individual studies, we included only studies that reported either confidence intervals or standard deviations. We performed meta-analysis of single means, choosing a random effects model to account for heterogeneity that is due to both random error and potential systematic differences between studies.^19^ The inverse variance method was used for pooling study estimates,^20^ and the DerSimonian-Laird estimator of tau-squared (a measure of variance representing between-study heterogeneity) was used to adjust standard errors.^21^ For confidence intervals around tau-squared and tau, the Jackson method was used.^22^ Forest plots were generated of the individual study means and confidence intervals alongside the pooled random effects estimates. The meta-analysis and figures were generated using R version 4.0.5 and the “meta” package version 4.18-1.^23,24^

Risk of bias assessment is meant to capture the quality of each study and the likelihood of producing biased results. To assess risk of bias in this review, we adapted criteria from the AHRQ Methods Guide for Comparative Effectiveness Reviews and Cochrane Handbook for Systematic Reviews of Interventions.^25,26^ Pairs of team members scored each study on sample selection, randomization process, protocol deviations, measurement of covariates, missing data, and presence of other potential biases. Scoring conflicts were resolved in consensus meetings. The score was then classified as low, moderate, or high risk of bias.

## RESULTS

As shown in Figure 1, the systematic literature search resulted in 406 studies in 4 subsets, focusing on verbal probabilities only, verbal and numerical probabilities, numbers and graphs only, and medication instructions. The current paper includes the first and second subsets: verbal probabilities only, and verbal versus numerical probabilities.

The final sample included 33 studies, which were published between 1967 and 2020 (Table 1). Of the studies, 14 were conducted in the UK, 12 in the USA, 5 in Australia, 1 in Canada, and 1 in Singapore. Fifteen focused on medication side effects, 14 on disease risks, and 4 with no specified context. Many studies provided little demographic information, with only 27 reporting participant gender, 25 age, 24 education, 5 ethnicity, and 4 socioeconomic status. Fourteen studies included actual patients, although primarily in hypothetical scenarios, while others recruited students, or members from the public (column 5 in Table 1). Most studies used relatively simple questionnaire study designs, and as a result most (64%, *n* = 21) had low or no risk of bias; 2 had high risk of bias.

**Table 1:** Articles on Verbal Probabilities and Characteristics Collected

Authors and year	Research question	Primary outcome measured	Sample size*	Sample description*	Health condition or situation	Severity of health event specified?	Study of EC† terms?	Risk of bias assessment
Lichtenstein and Newman 1967 ^27^	To assess numerical estimates and symmetry of interpretation of “mirror image” pairs of terms (e.g., “quite likely”-“quite unlikely”)	Numerical estimates	188	Adult males	Not specified	No	No	A little concern
Budescu et al. 1985 ^28^	To assess variability in the mapping of phrases to numbers	Numerical estimates	32	Faculty and graduate students of a university	Not specified	No	No	Moderate concern
Reagan et al. 1989^29^	To map verbal probability words to numbers	Numerical estimates	100	Undergraduate students	Not specified	No	No	Moderate concern
Shaw and Dear 1990^30^	To evaluate understanding of probability expressions and preference for receiving information	Numerical estimates, format preference	100	Adult female parents	Aspects of neonatal care	No	No	No concern
Weber and Hilton 1990^31^	To examine the role of context in the interpretation of probability words	Numerical estimates	85	Undergraduate students	Varying disease types and side effects	Some specified as severe life-threatening events, others unspecified	No	Moderate concern
Freeman et al. 1992^6^	To identify patients’ preferred risk language and physicians’ predictions about patient preferences	Format preference	208	Adult female patients with children from family practices	Vaccine risk	No	No	Moderate concern
Woloshin et al. 1994^32^	To assess patients’ interpretation of probability terms	Numerical estimates; format preference	307	Adult patients from a family practice	Medication side effect or complication risk from procedure	Minor vs major complications	No	Moderate concern
Hallowell et al. 1997 ^33^	To evaluate female patient preferences in formats used to present risk information during genetic counseling for breast and ovarian cancer	Format preference	43	Adult female patients presenting for genetic counseling in cancer clinic	Breast and ovarian cancer risks	No	No	A little concern
Franic et al. 2000^34^	To evaluate format preference in patient medication package inserts	Format preference	74	Adult female patients from academic university	Adverse drug reactions	No	No	High concern
Biehl et al. 2001^35^	To compare the interpretation of probability terms of adults with adolescents	Numerical estimates	34	Adults from a community center	Not specified	No	No	A little concern
Kaplowitz et al. 2002^36^	To evaluate how patients want, request, and receive cancer prognosis information	Format preference	352	Patients from the American Cancer Society (ACS) mailing list in Michigan, US	Cancer prognosis information	No	No	A little concern
Berry et al. 2002^37^	To assess the interpretation of verbal probability descriptors	Numerical estimates	268	Undergraduate and graduate students	Throat infection or ear infection; fictitious medication side effect	Mild vs severe side effects	Yes	Moderate concern
Berry et al. 2003^38^	To compare the understanding of verbal and numerical descriptions of medication side effects	Numerical estimates	360	Adults from various public settings	Fictitious medication side effect	Mild vs severe side effects	Yes	A little concern
Budescu et al. 2003^39^	To determine the directionality of probability phrases	Numerical estimates	27	Undergraduate students	Medical context; general medication administration	No	No	Moderate concern
Davey et al. 2003^40^	To evaluate women’s understanding of diagnostic test results	Numerical estimates	37	Adult women who had previously participated in a population survey	Breast cancer risk	No	No	A little concern
Lobb et al. 2003^41^	To evaluate how women wanted their risk of breast cancer to be described in consultation	Format preference	193	Adult women from cancer clinics	Breast cancer risk	No	No	Moderate concern
Berry et al. 2004^42^	To evaluate people’s interpretation of EC verbal descriptors for medication side effect risks	Numerical estimates	188	Adults from various public places	Over-the-counter painkiller medication side effects	No	Yes	A little concern
Berry et al. 2004^43^	To compare doctors’ and lay people’s interpretation of the EC verbal descriptors	Numerical estimates	134	Undergraduate and postgraduate students	Medication side effect	No	Yes	A little concern
Knapp et al. 2004^44^	To explore whether the EC verbal descriptors effectively convey the risk of side effects	Numerical estimates	120	Adults from cardiac rehabilitation clinics following a recent admission	Medication side effects for cardiac medication	No	Yes	A little concern
Berry and Hochhauser 2006^45^	To assess how verbal descriptors affect people’s perceptions of clinical trial participation risks	Numerical estimates	96	Adults from a train station	Fictional serious skin condition	No	No	A little concern
Hubal and Day 2006^46^	To evaluate the understanding of verbal probability terms and effects of alternative formats	Numerical estimates	222	Undergraduate students	Medication side effect	No	No	Moderate concern
Young and Oppenheimer 2006^47^	To assess how different formats of risk information influence medication compliance	Numerical estimates	120	Adult students from a university	Medication side effect	No	Yes	Moderate concern
France et al. 2008^48^	To compare the understanding of frequency of side effects when expressed in percentages or descriptive language	Numerical estimates, % correctly identified	50	Patients in the chest pain unit of an urban emergency department who had one or more ischemic heart disease factors	Risks of treatment for acute myocardial infarction	Severe vs less severe side effects	No	No concern
Graham et al. 2009^49^	To identify women’s preference and interpretation of language for description of the size of treatment complication risks	Format preference^‡^	262	Adult female patients undergoing routine follow-up visits for breast cancer	Breast cancer risk	No	No	A little concern
Knapp et al. 2009^50^	To assess the effectiveness of presenting side effect risk information in different formats	Numerical estimates	148	Adult users of an online cancer information website	Medication side effect	No	No	No concern
Nagle et al. 2009^51^	To evaluate female patients’ preference on risk of disease	Format preference	294	Adult female patients from a maternity unit	Down syndrome risk	No	No	No concern
Cheung et al. 2010^52^	To compare patients’ preference for risk presentation in medications	Format preference	240	Adult patients from arthritis clinics in a hospital and outpatient practice	Pain relief medication	No	Yes	A little concern
Vahabi 2010^53^	To evaluate whether format preference influences comprehension	Format preference	180	Adult female patients from various community settings	Breast cancer risk	No	No	High concern
Peters et al. 2014^54^	To measure risk comprehension and willingness to use a medication when presented with different formats	Numerical estimates	905	Adult participants from a paid online questionnaire	Cholesterol medication	No	Yes	No concern
Knapp et al. 2014^55^	To evaluate recommendations on communicating frequency information on side effect risk	Numerical estimates	339	Adult users of an online cancer information website	Medication side effects	No	No	A little concern
Webster et al. 2017^56^	To assess how people interpret the EC verbal descriptors	Numerical estimates, % correctly identified	1003	Adult users of an online survey conducted by a market research company	Medication side effects	Mild vs severe side effects	Yes	A little concern
Carey et al. 2018 ^57^	To assess patients’ interpretation of verbal descriptor chance of remission and preferences for format of risk communication	Numerical estimates, format preference	210	Adult medical oncology outpatients with a diagnosis of cancer	Cancer long-term side effects and chances of remission	No	No	A little concern
Wiles et al. 2020^58^	To determine the perceived risk of surgical complication risk using verbal probability terms	Numerical estimates	290	Adult patients attending a pre-operative assessment in a clinic	Major adverse postoperative complication	No	No	No concern

*Several studies contained both subsamples that met our inclusion criteria (adult laypeople) and other subsamples that did not (physicians, adolescents). As described in the “METHODS” section, these studies were included if the results for the eligible subsample were reported separately. For these studies, we report the sample size and sample description of the subgroup that met our inclusion criteria

†EC = European Community

‡Graham et al. 2009 ^49^ required respondents to choose from ordinal categories ranging from 1/100 to 1/10 000. The modal interpretation of “sometimes” was 1/100 (36% of women), “uncommon” 1/1000 (35%), “very uncommon” 1/10 000 (40%), “rare” 1/10 000 (58%) and “very rare” 1/10 000 (51%). Because of the categorical assessment and the fact that no larger numbers were provided to choose from, we did not average these results into the findings in Table 2*.*

**Figure 2:**
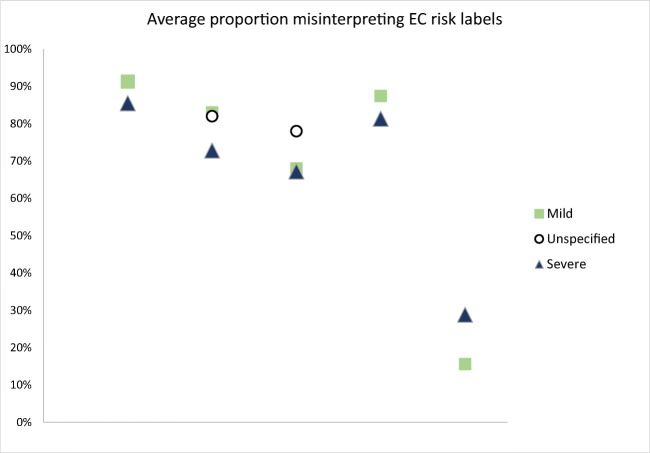
Average proportions misinterpreting European Commission (EC) risk labels across 2 studies. Legend: Among 2 large studies of EC verbal labels, including 1053 participants, an average of 70.1% misinterpreted the EC risk label. Rates of misinterpretation were similar whether the severity of the event was described or not, and if it was described, whether it was “mild” or “severe.” Misinterpretations were more common for more rare events, and there were only modest differences between interpretation of events described as “severe” versus “mild.”

Below, we present findings from the 2 subsets, which are not mutually exclusive: (1) 24 studies that elicited numerical estimates for verbal probability terms, including 9 focusing specifically on the EC terms (Table 2; Fig. 2), and (2) 11 studies (one of which contained 2 different samples) that assessed preferences for verbal versus quantitative risk information (Table 3).

Subset 1:

In 24 studies, numerical estimates were elicited for verbal probability terms. A total of 145 unique verbal probability terms were studied (Appendix 2). In some studies, the researchers also specified the severity of the event described by the verbal probability. We considered it likely that probability of mild outcome might be perceived differently than probability of a severe one. Therefore, we present these conditions separately, resulting in 14 unique probability-severity combinations (Table 2). Table 2 and Appendix 3 report pooled averages and ranges for 14 terms that were evaluated in at least three studies each and reported sufficient information for the meta-analysis. The term “rare” was estimated to mean a 10% risk, whereas the term “very likely” averaged 84%. Variability of interpretation of these terms was high, both across studies (minimum and maximum study averages reported in Table 2 column 5 and 6) and within study (ranges reported in column 7). For example, individuals estimated the term “rare” to mean anything from 0 to 80%, and “common” to mean anywhere between 10 and 100%. The effect of specifying the severity of the health event was modest. A “rare severe” event was judged slightly less likely than a “rare mild” event (10.1% versus 14.1% respectively), and a “common severe” event as slightly less likely than a “common mild” one (43.1% versus 50.5%). A subset of studies (9 indicated in Table 1) specifically examined interpretations of the EC probability labels. Meaningful summary data could not be generated for participant type (university student vs other adults) or information type (medication side effect vs procedure side effect vs disease risk) because samples in these subgroups were too small.

**Table 2:** Numeric Estimates of Verbal Probability Terms

Verbal probability term	Number of studies	Average numeric estimate, random effects model (%)	95% CI (%)	Minimum sample average (%)*	Maximum sample average (%)*	Range of individual estimates (%)^†^
Rare(ly)	7	10.00	[7.99, 12.01]	7.0	21	0–80
Rare-severe event	3	10.06	[5.45, 14.68]	6.3	34.8	–
Rare-mild event	3	14.14	[7.88, 20.40]	9.6	39.3	–
Uncommon	4	17.64	[13.19, 22.09]	13.3	22.9	0–90
Unlikely	6	17.71	[14.86, 20.55]	13.3	27	0–85
Common-severe event	3	43.08	[40.27, 45.88]	41.9	45.6	–
Possible(ly)	6	43.28	[36.66, 49.89]	36.9	62	–
Common-mild event	3	50.47	[45.59, 55.34]	48	58	–
Common	6	58.73	[50.40, 67.06]	34.2	70.5	10–100
Very common	3	60.10	[42.36, 77.85]	38.5	71.6	5–100
Probable(ly)	5	69.87	[67.07, 72.67]	66	73.9	20–100
Likely	6	71.87	[69.90, 73.84]	66	94	–
Usual(ly)	3	75.38	[71.53, 79.23]	72	78	–
Very likely	3	84.30	[79.43, 89.17]	75.2	93	20–100

Table includes terms studied in 3 or more studies in which sufficient information was reported for the meta-analysis

*All 19 studies reported an average estimate; minimum is the lowest of these averages, and maximum is the highest

†Only 4 studies reported ranges of estimates provided by individual participants within the study. This column reflects the range across all 4 studies

**Table 3:** Numbers and Proportions Preferring Verbal or Numeric Probabilities

Study *	Sample size	*n* (%)			
		Preferred verbal	Preferred numeric	Preferred combination	No preference
Woloshin et al. 1984^25^	307	91 (29.6)	135 (43.9)	81 (26.3)	NA
Shaw and Dear 1990^23^	81	43 (53.1)	30 (37.0)	NA	8 (9.9)
Freeman and Bass 1992^6^	208	89 (42.7)	119 (57.2)	NA	NA
Hallowell et al. 1997 ^33^	43	3 (7)	9 (21)	22 (52)	8 (19)
Franic and Pathak 2000^26^	74	4 (5.4)	70 (94.6)	NA	NA
Lobb et al. 2003 ^41^	109 (unaffected by condition)	24 (22.1)	55 (50)	20 (18.3)	10 (9.6)
	84 (affected by condition)	15 (17.9)	16 (19.2)	45 (53.8)	8 (9)
Graham et al. 2009 ^49^	262	136 (52)	125 (47.7)	NA	1 (0.3)
Nagle et al. 2009^38^	294	85 (28.9)	132 (44.9)	76 (25.8)	NA
Cheung et al. 2010^39^	240	60 (25.0)	180 (75.0)	NA	NA
Vahabi 2010^40^	180	61 (33.9)	119 (66.1)	NA	NA
Carey et al. 2018 ^57^	210	59 (28)	33 (16)	79 (38)	39 (18)

NA indicates that this option was not presented to respondents

*Kaplowitz et al. (2002)^36^ also evaluated format preference among cancer patients and survivors in a hypothetical choice between a verbal probability and a quantitative estimate of survival. However, the findings are not integrated into this table because the options were not mutually exclusive, and the authors do not clarify how many patients chose both. (Table 1 of that paper shows that 80% endorsed verbal probabilities and 53% endorsed quantitative information, suggesting that some subset must have chosen both)

In 2 of these studies, researchers additionally evaluated whether the participants misinterpreted the verbal probability term, defining misinterpretation as an estimate differing from the EC definition of the term (Fig. 2). As shown in Fig. 2, misinterpretation rates were higher for the terms indicating rare events, and there was relatively little difference between misinterpretation of the chances of mild events, severe events, and events of unspecified severity (Fig. 2).

Subset 2:

In 11 studies, participants’ preference for verbal versus numeric information was captured (one study contained 2 independently recruited samples for a total of 12 samples: Table 3). In 10 of the 12 samples, majorities (proportions ranging from 54 to 95%) preferred numeric risk information alone or in combination with verbal labels. In the 6 samples that had a choice between verbal, numeric, and combined formats, from 18 to 54% of respondents preferred the combination of numeric with verbal descriptions.

## DISCUSSION

Since 1967, 33 studies have examined lay interpretation of and preferences for verbal probability terms such as “rare” and “common” in health and medical contexts. These studies show that lay peoples’ numeric interpretations of these verbal terms are extremely variable and highly overlapping. For example, across the studies, individual participants estimated the term “rare” at anywhere between 0 and 80% probability, and the term “common” at between 10 and 100% probability. In other words, these studies provide no assurance that patients will perceive a health outcome described as “common” as more likely than one described as “rare.” This suggests that providers and health communicators should provide numbers where possible, avoiding situations in which words alone are used to describe risk.

In addition, the subset of studies examining the European Commission (EC) verbal terminology for risk in drug labels shows that these terms are usually misinterpreted and lead to numeric estimates far higher than the developers intended. For example, in the EC terminology, “rare” is intended to describe a risk between 0.1 and 0.01%, but the average lay interpretation was almost 10%, more than 100-fold higher. The EC term “common,” meant to describe a risk between 1 and 10%, was interpreted as an average of about 59%. It is clear that this verbal risk terminology is miscalibrated to lay perceptions, particularly for rare events. In particular, providers and health communicators who use the EC terms to describe chance of medication side effects should recognize that these terms are likely to vastly inflate perceptions of side effect risk.

The literature also suggests that majorities of patients prefer numeric risk information, alone or in combination with verbal labels. This finding suggests that healthcare professionals who choose verbal-only risk descriptors may not be meeting the preferences of their patients.

Overall, findings of these studies about health and medical risk communication are congruent with risk communication research in non-medical domains, which has similarly outlined the variability in interpretations of these terms.^10–13^

One limitation of our study stems from the search approach. It was challenging to create a literature search strategy for this problem because of the ubiquity of terms such as “risk” in non-communication domains such as epidemiology. We ended up calibrating the search strategy fairly broadly, requiring manual review to narrow down eligible articles. We did not restrict the search to a specific date range, and it is possible that language interpretation has changed over time. Another limitation is in the completeness of the research studies included, many of which did not provide details of demographics. We are therefore unable to draw conclusions from this review about the effects of education level, literacy, numeracy, socioeconomic status, race, or ethnicity on interpretations and information preferences. Other research has demonstrated that preference for numeric information is stronger among those with higher education or numeracy, and that individuals with lower levels of numeracy may express less comfort with numeric risk information.^59^ In addition, it is likely that numeracy would influence patients’ ability to assign a numeric probability to a verbal term.^3,60^ In focusing on the contrast between verbal and numeric risk information, we did not examine the vast literature on visualization and risk communication.

In summary, a systematic review of the literature provides strong evidence that patient interpretations of verbal probability terms are so variable that they may not distinguish between events of very different likelihoods. The evidence also suggests that most patients prefer numeric information about risks, either alone or in combination with verbal labels. These findings suggest that health professionals who avoid numbers by providing verbal probabilities alone are likely to have poor communication with their patients. Physicians and other healthcare professionals can improve the effectiveness of their communication with patients by providing accurate quantitative information about health risks.

## Supplementary Information

ESM 1 (DOCX 532 kb)

ESM 2 (DOC 63 kb)
