# Supplementary material for: Imprecision and Preferences in Interpretation of Verbal Probabilities in Health: a Systematic Review
Source: J Gen Intern Med. 2021 Aug 6;36(12):3820–9. doi: 10.1007/s11606-021-07050-7 (PMC8642516; doi:10.1007/s11606-021-07050-7)

**Appendix 1**

**Ovid MEDLINE** (ALL – 1946 to January 7, 2019)

Searched on January 9, 2019

No language, publication date, or study type restrictions

| **Line #** | **Search** |
| --- | --- |
| 1 | Multimedia/ |
| 2 | (animation or multimedia or multimedium).tw. |
| 3 | Audiovisual Aids/ or Webcasts as Topic/ |
| 4 | (Audio* or webcast* or podcast* or RSS or Really Simple Syndication).tw. |
| 5 | Videotape Recording/ or Video Games/ or Video Recording/ |
| 6 | (video or videos or videotape or videorecording or computer game* or visualization).tw. |
| 7 | Computer Graphics/ or Medical Illustration/ or Decision Trees/ |
| 8 | (bar chart* or pie chart* or drawing* or graphic* or graph or graphs or picture* or pictorial representation or pictogram or pictograph or illustration* or ornamentation or imprints or infographic or infogram or histogram or diagram or diagrams or icon or icons or visual representation* or data format or data presentation or risk communication).tw. |
| 9 | Anxiety/ |
| 10 | (anxiety or anxious or anxieties or nervousness or fear or concern or apprehension or worry).tw. |
| 11 | Comprehension/ |
| 12 | (comprehension or comprehending or understanding or readability).tw. |
| 13 | Decision Making/ or Choice Behavior/ |
| 14 | (decision making or decision satisfaction or decisional conflict or decisions or judgement or choice behavior or choice behaviour).tw. |
| 15 | Medication Adherence/ |
| 16 | ((medication or drug or dose or dosing or dosage) adj2 (adherence or nonadherence or noncompliance or non-adherence or persistence or compliance or non-compliance)).tw. |
| 17 | Medication Errors/ |
| 18 | ((medication or drug or dose or dosing or dosage) adj2 (error or errors)).tw. |
| 19 | (perceived effectiveness or perceived efficacy or perceived risk or perceived susceptibility or perceived usefulness).tw. |
| 20 | Mental Recall/ |
| 21 | recall.tw. |
| 22 | Trust/ |
| 23 | (trust or distrust or mistrust or trustworthiness).tw. |
| 24 | Emotions/ |
| 25 | (emotional response or emotional factor or expressed emotion).tw. |
| 26 | Mathematical Concepts/ |
| 27 | (mathematical concept* or numeracy or numeric or numbers or numeral or numerical or numerosity or quantitative data or quantitative information or quantitative literacy or statistical information or statistical literacy or statistical interpretation or statistical data or natural frequency or natural frequencies or risk comprehension or risk interpretation or risk reduction).tw. |
| 28 | 26 or 27 |
| 29 | or/1-8 |
| 30 | or/9-25 |
| 31 | 28 and 29 and 30 |

**Appendix 2: Full list of terms studied**

This Appendix includes the complete list of terms studied in any study. Many of these terms were studied in only 1 or 2 papers, and most did not include the variance estimates required to perform metaanalysis. Therefore, for illustrative purposes only, we have computed the simple weighted average as a way of ranking the terms from lowest estimate to highest. It should be noted that this approach is less rigorous than the metaanalysis presented in the main paper.

| **Verbal Probability Term** | **Number of Studies** | **Average estimate, weighted (%)** | **Minimum of Averages (%)** | **Maximum of Averages (%)** | **Range** |
| --- | --- | --- | --- | --- | --- |
| A Minority Of | 1 | 20.50 | -- | -- | -- |
| A Significant Proportion | 1 | 70.70 | -- | -- | -- |
| Almost Certain(ly) | 2 | 87.40 | 84.80 | 90.00 | 35-100 |
| Almost Impossible | 1 | 2.00 | -- | -- | -- |
| Almost Never | 1 | 9.90 | -- | -- | -- |
| Always | 2 | 94.16 | 94.00 | 94.32 | -- |
| As Often as Not | 1 | 50.97 | -- | -- | -- |
| Barely Possible | 1 | 13.00 | -- | -- | 1-60 |
| Better Than Even | 1 | 58.00 | -- | -- | 45-89 |
| Can | 1 | 48.50 | -- | -- | -- |
| Causes | 1 | 82.40 | -- | -- | -- |
| Certain | 1 | 91.50 | -- | -- | -- |
| Common | 10 | 58.60 | 34.20 | 70.50 | 10-100 |
| Common-Mild | 4 | 53.60 | 48.00 | 58.00 | -- |
| Common-Severe | 4 | 43.60 | 41.90 | 45.60 | -- |
| Definitely | 0 | NA | -- | -- | 50-100 |
| Definitely Not | 0 | NA | -- | -- | 0-10 |
| Develop | 1 | 53.40 | -- | -- | -- |
| Even Chance | 1 | 50.00 | -- | -- | -- |
| Experience | 1 | 50.60 | -- | -- | -- |
| Faintly Possible | 1 | 13.00 | -- | -- | 1-60 |
| Fair Chance | 1 | 51.00 | -- | -- | 20-85 |
| Fairly Likely | 1 | 66.00 | -- | -- | 15-95 |
| Fairly Unlikely | 1 | 25.00 | -- | -- | 2-75 |
| Feel | 1 | 48.60 | -- | -- | -- |
| Few | 1 | 15.80 | -- | -- | -- |
| Fighting Chance | 1 | 47.00 | -- | -- | 5-90 |
| Frequent(ly) | 3 | 71.47 | 69.60 | 73.8 | -- |
| Good Chance | 1 | 74.00 | -- | -- | 25-96 |
| Have | 1 | 51.60 | -- | -- | -- |
| High Chance | 2 | 82.53 | 80.00 | 85.06 | -- |
| High Probability | 1 | 85.21 | -- | -- | -- |
| High-Mild | 1 | 60.50 | -- | -- | -- |
| High-Severe | 1 | 40.70 | -- | -- | -- |
| Highly Improbable | 1 | 6.00 | -- | -- | 1-30 |
| Highly Probable | 1 | 89.00 | -- | -- | 60-99 |
| Improbable | 5 | 12.70 | 6.00 | 17.00 | -- |
| Include | 1 | 67.00 | -- | -- | -- |
| Inconclusive | 1 | 43.00 | -- | -- | 1-75 |
| Infrequent | 1 | 16.20 | -- | -- | -- |
| Is Associated With | 1 | 69.40 | -- | -- | -- |
| Less | 1 | 31.80 | -- | -- | -- |
| Less Often Than Not | 1 | 35.53 | -- | -- | -- |
| Likely | 10 | 72.10 | 66.00 | 94.00 | 15-100 |
| Low Chance | 2 | 17.19 | 14.28 | 20.00 | -- |
| Low Probability | 1 | 17.94 | -- | -- | -- |
| Low-Mild | 1 | 17.20 | -- | -- | -- |
| Low-Severe | 1 | 14.60 | -- | -- | -- |
| Manifested | 1 | 65.50 | -- | -- | -- |
| Many | 1 | 69.90 | -- | -- | -- |
| May | 1 | 47.20 | -- | -- | -- |
| Medium Chance | 1 | 50.00 | -- | -- | -- |
| Might | 1 | 43.10 | -- | -- | -- |
| Minimal-Mild | 1 | 9.70 | -- | -- | -- |
| Minimal-Severe | 1 | 8.70 | -- | -- | -- |
| Moderate Chance | 1 | 51.29 | -- | -- | -- |
| Moderate Probability | 1 | 38.71 | -- | -- | -- |
| Moderate-Mild | 1 | 34.40 | -- | -- | -- |
| Moderate-Severe | 1 | 23.80 | -- | -- | -- |
| More | 1 | 65.80 | -- | -- | -- |
| More Often Than Not | 1 | 56.76 | -- | -- | -- |
| Most | 1 | 79.40 | -- | -- | -- |
| Negligible-Mild | 1 | 8.50 | -- | -- | -- |
| Negligible-Severe | 1 | 7.40 | -- | -- | -- |
| Never | 2 | 4.78 | 0.00 | 9.56 | -- |
| Not Improbable | 2 | 56.50 | 50.00 | 63.00 | -- |
| Not Likely | 2 | 17.50 | 11.00 | 24.00 | -- |
| Not Much Chance | 1 | 16.00 | -- | -- | 1-45 |
| Not Often | 1 | 17.76 | -- | -- | -- |
| Not Quite Even | 1 | 44.00 | -- | -- | -- |
| Not Very Often | 1 | 22.62 | -- | -- | 5-60 |
| Not Very Probable | 1 | 20.00 | -- | -- | 1-60 |
| Noted | 1 | 35.00 | -- | -- | -- |
| Observed | 1 | 29.40 | -- | -- | -- |
| Occasionally | 1 | 47.00 | -- | -- | 20-100 |
| Occurs | 1 | 65.40 | -- | -- | -- |
| Often | 2 | 71.40 | 69.79 | 73.00 | -- |
| Possible(ly) | 6 | 39.10 | 36.90 | 62.00 | -- |
| Predictable | 2 | 68.50 | 63.00 | 74.00 | -- |
| Pretty Good Chance | 1 | 67.00 | -- | -- | 25-95 |
| Probable(ly) | 6 | 68.90 | 66.00 | 73.90 | 20-100 |
| Produces | 1 | 84.70 | -- | -- |  |
| Quite Improbable | 2 | 10.50 | 3.00 | 18.00 |  |
| Quite Likely | 3 | 81.40 | 79.00 | 94.00 |  |
| Quite Unlikely | 1 | 11.00 | -- | -- | 1-50 |
| Rare(ly) | 8 | 9.70 | 7.00 | 21.00 | 0-80 |
| Rare-Mild (0.02%) | 4 | 19.20 | 9.60 | 39.30 |  |
| Rare-Severe (0.02%) | 4 | 14.80 | 6.30 | 34.80 |  |
| Rather | 1 | 58.00 | -- | -- | 10-80 |
| Rather Likely | 1 | 69.00 | -- | -- | 15-99 |
| Rather Unlikely | 1 | 22.50 | -- | -- | 1-67.5 |
| Reported | 1 | 36.70 | -- | -- |  |
| Seldom | 2 | 16.00 | 16.00 | 16.00 |  |
| Several | 1 | 44.60 | -- | -- |  |
| Shown | 1 | 37.90 | -- | -- |  |
| Slightly Less Than Half the Time | 1 | 45.00 | -- | -- | 5-50 |
| Slightly More Than Half the Time | 1 | 55.00 | -- | -- | 45-80 |
| Slights Odds Against | 1 | 45.00 | -- | -- | 10-99 |
| Slights Odds in Favor | 1 | 55.00 | -- | -- | 5-75 |
| Some | 2 | 23.85 | 14.70 | 33.00 |  |
| Sometimes | 2 | 39.65 | 29.30 | 50.00 | 20-100 |
| Somewhat | 1 | 52.20 | -- | -- | -- |
| Somewhat Improbable | 2 | 28.00 | 25.00 | 31.00 | -- |
| Somewhat Likely | 3 | 61.00 | 59.00 | 63.00 | 20-92 |
| Somewhat Unlikely | 1 | 31.00 | -- | -- | 3-80 |
| Toss Up | 2 | 48.50 | 47.00 | 50.00 | -- |
| Uncertain | 2 | 40.50 | 40.00 | 41.00 | 8-90 |
| Uncommon | 4 | 16.60 | 13.30 | 22.90 | 0-90 |
| Uncommon-Mild | 2 | 20.30 | 20.30 | 20.30 | -- |
| Uncommon-Severe | 2 | 14.50 | 14.50 | 14.50 | -- |
| Unlikely | 6 | 15.90 | 13.30 | 27.00 | 0-85 |
| Unpredictable | 1 | 42.00 | -- | -- | -- |
| Usual(ly) | 3 | 74.60 | 72.00 | 78.00 | -- |
| Usually Not | 1 | 18.00 | -- | -- | -- |
| Very | 1 | 79.00 | -- | -- | -- |
| Very Common | 3 | 59.90 | 38.50 | 71.60 | 5-100 |
| Very Common-Mild | 2 | 67.20 | 67.20 | 67.20 | -- |
| Very Common-Severe | 2 | 64.00 | 64.00 | 64.00 | -- |
| Very Good Chance | 1 | NA | -- | -- | 20-100 |
| Very High Chance | 2 | 89.47 | 88.94 | 90.00 | -- |
| Very High Probability | 1 | 87.26 | -- | -- | -- |
| Very Improbable | 3 | 12.00 | 5.00 | 22.00 | -- |
| Very Likely | 6 | 86.00 | 75.20 | 93.00 | 20-100 |
| Very Low Chance | 2 | 10.36 | 10.00 | 10.71 | -- |
| Very Low Probability | 1 | 9.74 | -- | -- | -- |
| Very Low-Mild | 1 | 10.70 | -- | -- | -- |
| Very Low-Severe | 1 | 10.10 | -- | -- | -- |
| Very Often | 1 | 77.79 | -- | -- | -- |
| Very Possible | 1 | 80.00 | -- | -- | -- |
| Very Probable | 2 | 83.50 | 80.00 | 87.00 | 60-99 |
| Very Rare | 1 | 9.60 | -- | -- | -- |
| Very Rare-Mild | 2 | 4.90 | 4.90 | 4.90 | -- |
| Very Rare-Severe | 2 | 2.90 | 2.90 | 2.90 | -- |
| Very Unlikely | 3 | 10.00 | 9.00 | 15.10 | 0-30 |
| Will Certain Happen | 1 | 90.76 | -- | -- | -- |
| Will Certainly Not Happen | 1 | 11.65 | -- | -- | -- |
| Will Definitely Happen | 1 | 97.09 | -- | -- | -- |
| Will Definitely Not Happen | 1 | 9.09 | -- | -- | -- |
| Negligible Risk | 1 | 5.00 | -- | -- | -- |
| Minimal Risk | 1 | 5.00 | -- | -- | -- |
| Low Risk | 1 | 10.00 | -- | -- | -- |
| Standard Risk | 1 | 20.00 | -- | -- | -- |
| Moderate Risk | 1 | 33.00 | -- | -- | -- |
| High Risk | 1 | 70.00 | -- | -- | -- |
| Very High Risk | 1 | 90.00 | -- | -- | -- |

**Appendix 3: Forest plots for 14 terms presented in Table 2**


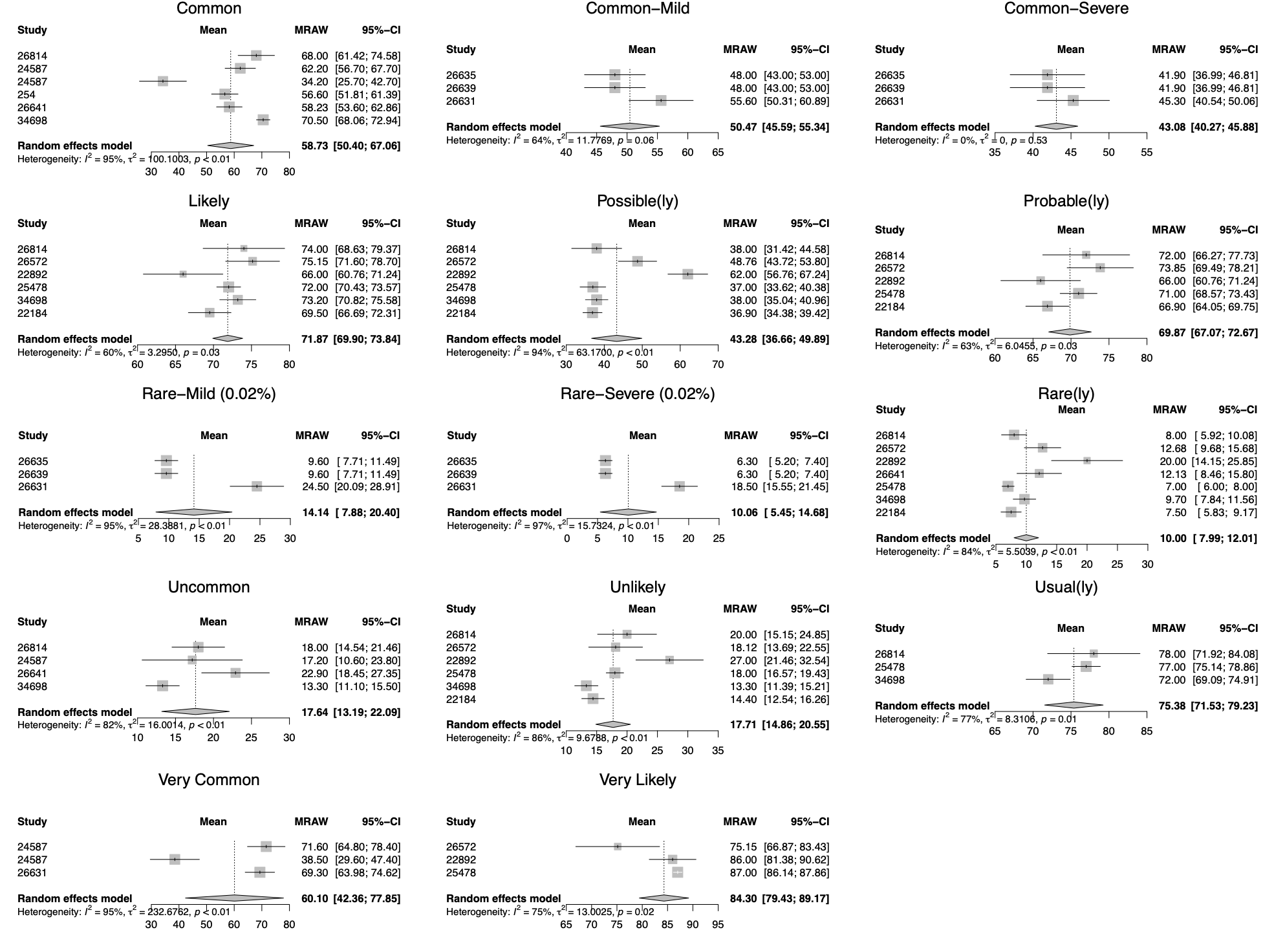

Supplement: Supplementary file 1 — (DOCX 532 kb) [file 11606_2021_7050_MOESM1_ESM.docx]
